# mHealth App Prescription in Australian General Practice: Pre-Post Study

**DOI:** 10.2196/16497

**Published:** 2020-06-01

**Authors:** Oyungerel Byambasuren, Elaine Beller, Tammy Hoffmann, Paul Glasziou

**Affiliations:** 1 Institute for Evidence-Based Healthcare Bond University Robina Australia

**Keywords:** mHealth apps, app prescription, general practice

## Abstract

**Background:**

Evidence of effectiveness of mobile health (mHealth) apps as well as their usability as non-drug interventions in primary care are emerging around the globe.

**Objective:**

This study aimed to explore the feasibility of mHealth app prescription by general practitioners (GPs) and to evaluate the effectiveness of an implementation intervention to increase app prescription.

**Methods:**

A single-group, before-and-after study was conducted in Australian general practice. GPs were given prescription pads for 6 mHealth apps and reported the number of prescriptions dispensed for 4 months. After the reporting of month 2, a 2-minute video of one of the apps was randomly selected and sent to each GP. Data were collected through a prestudy questionnaire, monthly electronic reporting, and end-of-study interviews. The primary outcome was the number of app prescriptions (total, monthly, per GP, and per GP per fortnight). Secondary outcomes included confidence in prescribing apps (0-5 scale), the impact of the intervention video on subsequent prescription numbers, and acceptability of the interventions.

**Results:**

Of 40 GPs recruited, 39 commenced, and 36 completed the study. In total, 1324 app prescriptions were dispensed over 4 months. The median number of apps prescribed per GP was 30 (range 6-111 apps). The median number of apps prescribed per GP per fortnight increased from the pre-study level of 1.7 to 4.1. Confidence about prescribing apps doubled from a mean of 2 (not so confident) to 4 (very confident). App videos did not affect subsequent prescription rates substantially. Post-study interviews revealed that the intervention was highly acceptable.

**Conclusions:**

mHealth app prescription in general practice is feasible, and our implementation intervention was effective in increasing app prescription. GPs need more tailored education and training on the value of mHealth apps and knowledge of prescribable apps to be able to successfully change their prescribing habits to include apps. The future of sustainable and scalable app prescription requires a trustworthy electronic app repository of prescribable mHealth apps for GPs.

## Introduction

More than 350,000 apps exist in the Medical and Health and Fitness categories in major app stores [1], with downloads and revenues in the billions [2]. Their popularity and potential to influence health-related behaviors make their integration to medical practice imminent [3]. Pragmatic studies of app prescription in primary care have been emerging around the world with varied interventions and results [4-6]. To assist the integration of apps into clinical practice, mobile health (mHealth) app repositories have been created, including the National Health Service App library in the United Kingdom [7], Health Navigator in New Zealand [8], and other private entities such as AppScript [9] and the Organization for the Review of Care and Health Applications [10].

Given the potential of mHealth apps to help improve the self-management of chronic conditions, we explored their value in general practice. Previously, in an overview of systematic reviews, we explored the possibility of simple integration of mHealth apps into the general practice setting and proposed a concept of “prescribable” mHealth apps. These were defined as proven effective (that is, shown to help achieve measurable clinical improvements in patients’ conditions), in addition to being standalone and currently available in the app stores [11].

We also explored the potential barriers to app integration in Australian general practice [12]. Patients expressed their preference for doctor-recommended apps; however, doctors were overwhelmed by the sheer number of available apps and faced 2 major barriers: not knowing of many prescribable apps and the lack of trustworthy source to access such apps. To address these barriers, we developed a brief implementation intervention. Objectives of this study were to explore the feasibility of app prescription by general practitioners (GPs) and to evaluate the effectiveness of an implementation intervention to increase uptake of app prescription.

## Methods

### Study Design and Setting

We employed a single-group, before-and-after design. Our study was conducted in the Australian general practice setting. Ethics approval was obtained from the Bond University Human Research Ethics Committee (#OB00017).

### Participants and Recruitment

GPs currently working in Australia at least 2 days a week were eligible to participate in our study. Information about the study was distributed at 2 annual national GP conferences (GPDU2018 and GP18) and posted to a closed Facebook group called GPs DownUnder. Recruitment occurred from June 2018 through November 2018, and data collection occurred from September 2018 until May 2019. Upon completion of the study, GPs were thanked with Aus $50 gift cards.

### Intervention

**Figure 1:**
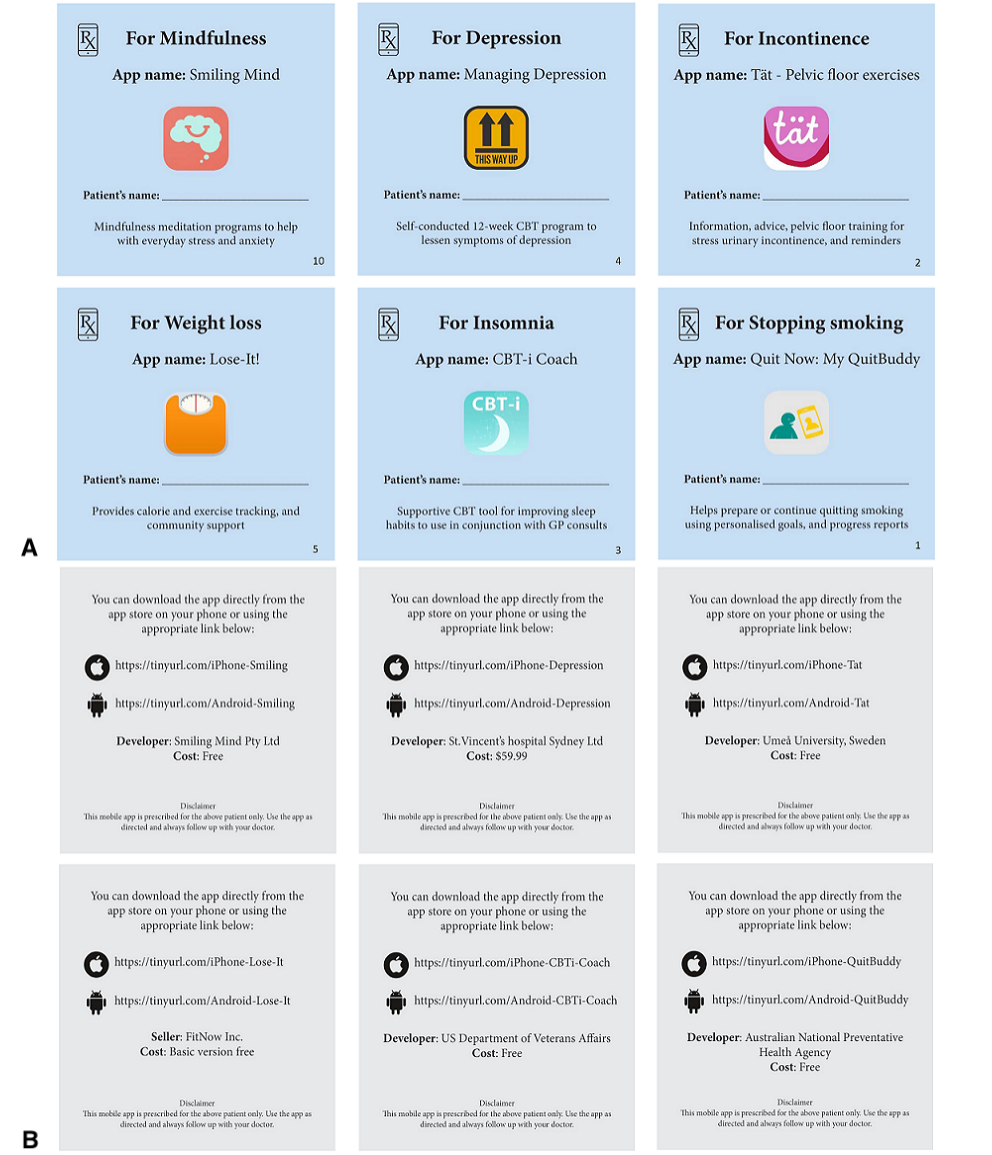
The 6 app prescription pads, showing the front (A), with prescription details and script number in the bottom right corner, and back (B), with app download instructions and cost.

There were two parts to the intervention. First, prescription pads for 6 apps were developed (Figure 1). These apps were chosen because they address conditions relevant in general practice, have either direct trial evidence (This Way Up: Managing Depression, St. Vincent’s Hospital Sydney Ltd [13]; Tät – Pelvic floor exercises, Umeå University, Sweden [14]; Lose-It!, FitNow Inc [15,16]; CBT-i Coach, US Department of Veteran’s Affairs [17]) or indirect evidence from trials of similar apps (Smiling Mind, Smiling Mind Pty Ltd [18-20] and Quit Now: My QuitBuddy, Australian National Preventative Health Agency [21]). The apps also had to have stable content, were created or backed by trustworthy not-for-profit organizations, and were available for both Apple and Android phones. Five of the apps were freely available, and one (This Way Up: Managing Depression) had a one-time purchase price of Aus $59.99. The cost of apps was not an exclusion criterion as it will help assess if cost is a barrier to app prescription.

The app prescription pads had individually numbered pages with a tear-off design. Each app prescription page included the app’s full name and logo, download instructions, space for the patient’s name, the reason for prescription, and a disclaimer. Prescription pads were assembled onto an A4 display stand and mailed to participating GPs. A letter outlining the study timelines and procedures along with a short introduction to each app was included in the shipment.

The second part of the intervention was aimed at enhancing uptake. Short videos (2 minutes) demonstrating the content, functions, and features of the apps in detail were created for each app. A YouTube link to the video randomly selected for each participant was emailed following their second month’s reporting.

Our study aimed to change the prescribing behavior of GPs. Evidence suggests that behavioral interventions are more effective and sustainable when guided by behavior change techniques. Our prior research helped to identify the target behaviors [12]. We based our intervention on the Capability, Opportunity, Motivation, and Behavior model [22]. Capability to prescribe apps was addressed through the list of evidence-based apps and the introductory videos demonstrating the content, features, and function of the apps; opportunity was enabled through the purposefully designed stand with the prescription pads; and motivation was harnessed through the GPs’ expressed interest in the study that demonstrates their belief that app prescription would be a good thing to do [23].

### Procedures

At the beginning of the study, participants signed consent forms and answered the prestudy questionnaire via the web-based SurveyMonkey tool (SurveyMonkey Inc, San Mateo, CA). The survey collected demographic data, contact details, current app prescription rate in the preceding 2 weeks (self-reported in ranges: 0, 1-5, 6-10, >10 times), and level of confidence around app prescription.

The official commencement dates for the study were recorded as the date that each participant reported they started using the pads. Every 4 weeks following commencement, participants were asked to send a photo of the prescription pads electronically to the research team to provide details of the number of prescriptions for each app within that month. If participants took leave from work, the reporting dates were adjusted to allow for a full 4-week reporting period.

Qualitative semistructured interviews (10-15 minutes) were conducted and audio recorded at the end of the study, either face-to-face or by telephone, to gather feedback on the intervention. GPs were asked about their knowledge of other apps and relevant resources outside the study, including the Handbook of Non-Drug Interventions (HANDI) project by the Royal Australian College of General Practitioners, which includes a number of mHealth apps. Interviews were transcribed verbatim, coded by the lead researcher (OB), and thematically analyzed to determine the feasibility of the interventions, barriers, and solutions to the scalability of the intervention to Australian GPs. The thematic analysis was done in consultation with a second author (TH).

### Sample Size

Prior data [12] indicated that the difference in the response before and after is normally distributed with a standard deviation of 10 and a baseline mean of 2 apps prescribed per month per GP. We calculated that we needed 24 participants to have 80% power (with α=.05) to detect an increase of app prescription by at least 6 per month. Taking attrition into account, we planned to recruit 30 GPs for the study.

### Outcomes

Data on app usage were collected for the 2-week period prior to study commencement and then every month for 4 months. The primary outcome of the study was the number of app prescriptions dispensed in total, as an average per month, per GP, and per GP per fortnight. We calculated the median number of apps recommended by a GP per fortnight using the following formula:

m=l + (w(n/2-c))/f

where l is the lower limit of the bin (range) containing the median, w is the width of the bin, n is the total population, c is the cumulative count (frequency) up to l, and f is the count in the median bin.

Prestudy raw numbers are provided in Table 1 (m=1.7 [1 + (5(39/2-17))/19]). Poststudy numbers are given in the Results section (m=4.1 [1 + (5(39/2-0))/31]).

Secondary outcomes were confidence around prescribing apps (measured on a 5-point Likert scale; prestudy and poststudy); the number of intervention video views and their impact on the subsequent prescription numbers; and attrition rate. In addition, the acceptability of the interventions to GPs and their feedback on the interventions were explored in semistructured interviews. Descriptive statistics were used to report the frequency of app use at each time point and confidence in app prescription. Qualitative data were analyzed thematically.

To conduct the overall analysis of the effect of video exposure on prescription rates, the 6 separate outcomes (1 for each app) were considered as one overall global outcome (individual monthly counts were not aggregated). Initially, a Poisson model was fitted with the (categorical) explanatory variables specified: the month (1 to 4), exposure to the video (yes/no), video (1 to 6), and interaction between exposure and video. To account for the 24 repeated measures collected for each GP (4 timepoints by 6 apps), a random intercept was fitted. Overdispersion was assessed using generalized chi-square divided by degrees of freedom. Due to evidence of overdispersion for the Poisson model (generalized chi-square/degrees of freedom=2.13), a negative binomial model was fitted and showed no evidence of over-dispersion (generalized chi-square/degrees of freedom=0.98).

## Results

### Overview

A total of 40 currently practicing Australian GPs were recruited for this study. One GP dropped out before the beginning of the study, and 3 GPs dropped out after the second and third data collection due to relocation and change of jobs. The full 4-month study was completed by 36 GPs, and we analyzed the data as intention-to-treat (ITT). The median age of the participants was 40 years, the median length of time in practice was 8.5 years, and participants worked a median of 4 days a week (Table 1).

**Table 1:** Participant demographics and prestudy characteristics, n=39.

Characteristics		n (%)
**Age (years)**		
	≤35	11 (28)
	36-45	20 (51)
	46-55	5 (13)
	≥56	3 (8)
**Years in practice**		
	≤10	23 (59)
	11-20	12 (31)
	≥21	4 (10)
Female gender		28 (72)
**Geographical distribution**		
	Queensland	21 (54)
	New South Wales	9 (23)
	Victoria	4 (10)
	South Australia	2 (5)
	Western Australia	2 (5)
	Tasmania	1 (3)
**Days worked in a week**		
	2	8 (21)
	3	9 (23)
	4	13 (33)
	≥5	9 (23)
**Number of apps prescribed in the 2 weeks prior to the study**		
	0	17 (44)
	1-5	19 (49)
	6-10	3 (8)
**Confidence level with app prescribing**		
	Not at all (1)	7 (18)
	Not so (2)	12 (31)
	Somewhat (3)	19 (49)
	Very (4)	1 (3)
	Extremely (5)	0

### Prescriptions

**Figure 2:**
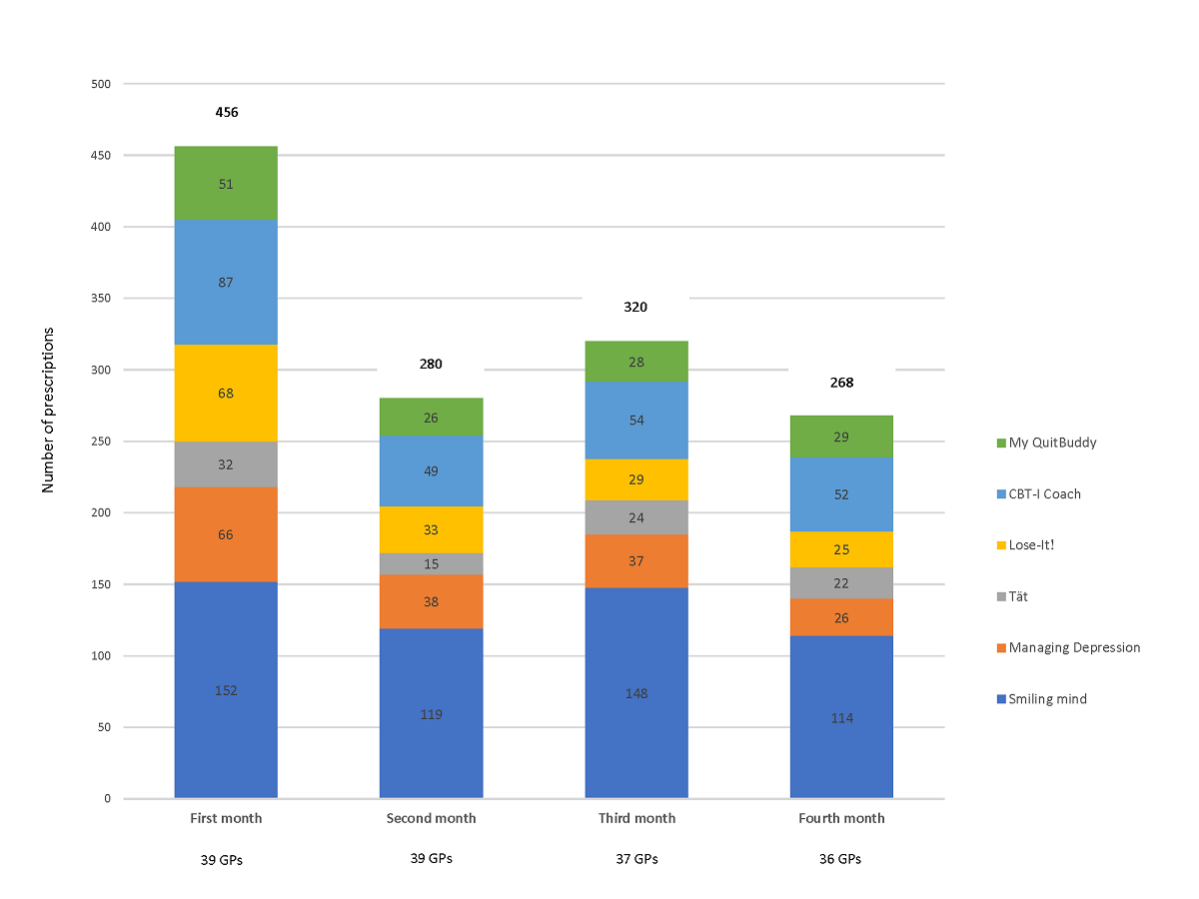
Number of individual app prescriptions shown in the monthly totals.

In total, 1324 app prescriptions were dispensed over 4 months, with a mean of 331 prescriptions a month. Figure 2 illustrates the number of individual app prescriptions within the monthly totals. The number of apps prescribed per GP per fortnight increased from an imputed prestudy median of 1.7 to 4.1. Overall, the Smiling mind app was the most frequently prescribed (533/1324, 40%), followed by CBT-i Coach (242/1324, 18%), Managing Depression (167/1324, 13%), Lose-It! (155/1324, 12%), Quit Now: My QuitBuddy (134/1324, 10%), and Tät Pelvic floor exercises (93/1324, 7%).

**Figure 3:**
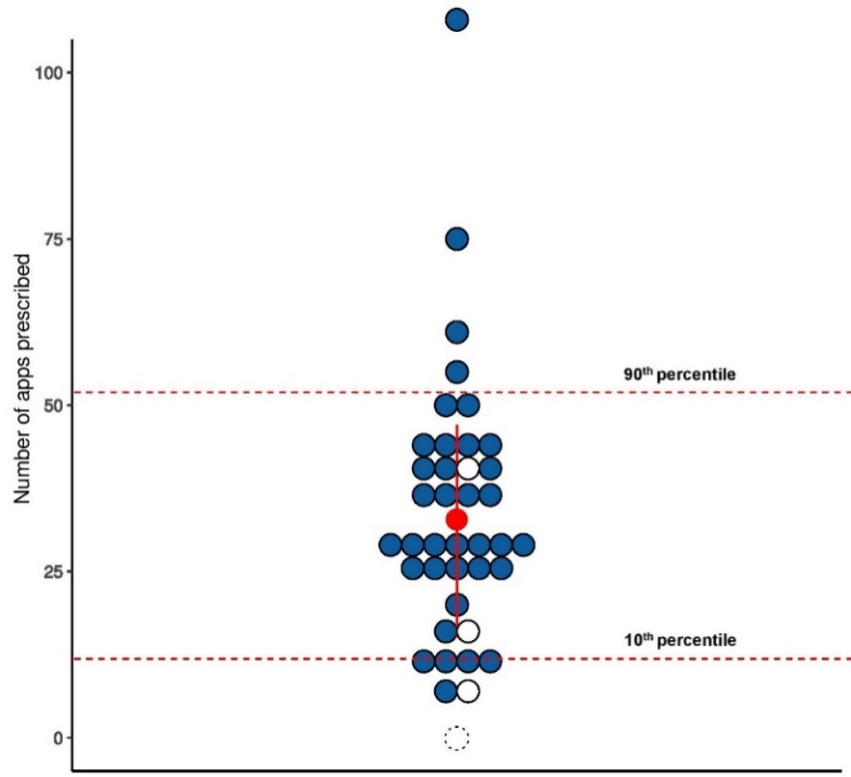
Distribution of total app prescription per general practitioner (GP). The red dot indicates the median (30 apps), the white dots indicate the participants who dropped out, and the dashed circle represents the participant who never commenced.

Figure 3 illustrates the distribution of the total app prescription per GP. According to the ITT analysis, a median of 30 apps (range 6-111 apps) was prescribed per GP over the 4 months. Every GP prescribed at least one app per fortnight, 31 (31/39, 80%) GPs prescribed 1-5 apps, 7 (7/39, 18%) prescribed 6-10 apps, and 1 GP prescribed more than 11 apps. The GPs’ confidence around prescribing apps doubled from a mean of 2 (not so confident) before the study to 4 (very confident) at the end of the study: 0/39 not confident at all; 1/39 (3%) not so confident; 12/39 (31%) somewhat confident; 25/39 (64%) very confident; 1/39 (3%) extremely confident.

**Figure 4:**
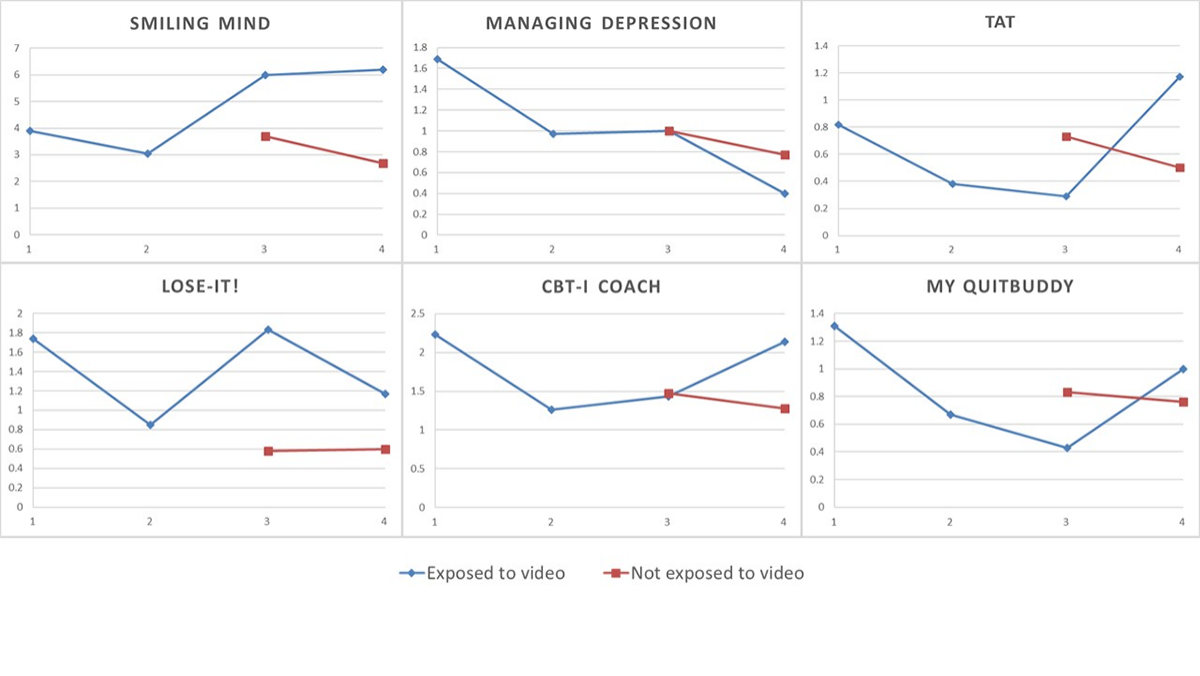
Mean number of app prescriptions per general practitioner (GP) before and after exposure to the intervention video in each month.

At the end of the study, the My QuitBuddy app video was viewed 8 times; the Smiling mind, Managing Depression, and Lose-It! app introduction videos were viewed 9 times each; the Tat-Pelvic floor exercise video was viewed 19 times; and the CBT-i Coach video was viewed 21 times. We were not able to track whether every GP watched the video sent to them. The effects of exposure to app videos are shown in Figure 4. Only two of the app videos had a significant effect on the subsequent app prescription numbers following the exposure to the video: Smiling Mind app prescription increased from 3-4 times per month to 6 times per month, and Lose-It! app prescription increased by one time. The full analysis is provided in Multimedia Appendix 1. A global test for the interaction between exposure and video showed strong evidence of heterogeneity (*P*<.001) indicating the treatment effects were different across the 6 apps. Therefore, we did not report an overall effect of the videos.

### Qualitative Interviews

As per the ITT analysis, 39 GPs were interviewed at the conclusion of their participation in the study. Participants expressed their overall experience of prescribing apps as overwhelmingly positive. They liked the size of the prescription pad, the information included on it, ease of use, and integration into the workflow, with the most useful feature identified as the visual cue aspect of the stand. They also liked the short length of the videos, yet felt they contained sufficient details about the apps. Most GPs reported not downloading and interacting with the apps themselves. Although most reported having watched the allocated video, many did not recall the contents during the poststudy interviews.

Two of the 6 study apps were well known to the GPs: 28/39 GPs were already familiar with Smiling Mind, and 12/39 GPs were already familiar with Managing Depression. They had been recommending these apps to their patients even before the study and appreciated having a formal prescription to give out during the study. Among the other apps that GPs recommended, mindfulness and meditation apps (Calm, Headspace) were common. Mental health–related apps were the most frequently prescribed, and all GPs reported that the overall number of apps they prescribed is a reflection of the demographics of their patients and the prevalence of conditions encountered.

GPs reported that they might have prescribed the weight loss and pelvic floor exercise apps more frequently. Instead, they habitually referred patients to dietitians and physiotherapists or to programs and tools already compiled as the first line of intervention. None of the GPs, except for one, had watched, read, or received any other app-related content apart from the study intervention. Knowledge of HANDI was low, especially that apps were included in some HANDI entries. However, upon learning this, they all agreed that HANDI would be a reliable evidence-based app repository for GPs in Australia. The main barriers and facilitators to app prescription in general practice are shown in Table 2 along with illustrative quotes.

**Table 2:** Key themes and illustrative quotes around barriers and facilitators of mobile health app prescription in general practice.

Theme type, theme		Illustrative quotes
**Barriers**		
	Poor knowledge and familiarity of prescribable apps	“I think from a doctor, it's purely just knowledge of health apps.”
		“From the GP^a^ point of view, thinking about it, knowing which ones are good and which ones aren't.”
		“challenging because I wasn't necessarily familiar myself with the details of the app in terms of using them myself or actually being able to really coach patients with using them. I guess that takes time to sit down and actually go through the apps.”
	Prescribing habit	“Getting into the habit of having those things available was part of the process, trying to trigger the idea that I can do this was part of it.”
		“I think trying to, in a busy consultation, trying to remember that as an option that we could recommend to people, because often you're so busy going, here, have this, do this, have this medication and then you often - adding some sort of self-help app into this is just part of getting more used to thinking about it as being an option.”
	Cost of apps	“The depression one was quite an expensive app, that was quite prohibitive to a lot of people.”
		“I guess I think cost definitely is a barrier for some patients, especially those that are in financial difficulty because they even ask for a referral to a bulk-billing psychologist.”
	Patients’ capability and attitude towards mHealth^b^ apps	“I think they're probably for me the two big factors, is (one, the doctor's knowledge of them and) two, the patient perception of how important it is or the value of these health apps in terms of part of their management plan.”
		“A lot of my main issue was the demographic of my patients. I didn't realize how much I would struggle to incorporate it because I actually have a huge percentage of elderly patients who don't even have smartphones and some of them that do, don't know how to use the apps properly.”
	Consultation time	“time constraints, a lot of the time we're running behind and the app prescription is a slightly luxury, but when we have time and we're able to be thorough, of course, we can do it, but we don't always have that luxury of time”
		“Time is just such a big issue because we're so time-pressured.”
**Facilitators**		
	Tailored education, face-to-face training, and information dissemination to increase knowledge of prescribable apps	“it's one message consistent and persistent. So if you've got a list that you're confident in, then why are you confident in it, what's the message behind and then you get it out as many ways as you can because none of us is looking at everything all the time. So if there's some way to get it out to the colleges, is there some way to get it out of the journals, is there somewhere to put it online somewhere that's an authoritative source, is there some way to get it out through the universities? Word of mouth is always good, influencers, social media…
		“Coming and meeting us and going through face to face, maybe demonstrating some, a bit like the drug reps do”
		“I mean getting doctors early, so getting them through their training programs, getting them as GP registrars and making it part of there, I think that's where you're going to really get significant change.”
	Meaningful familiarity with apps	“GP's own familiarity with the app, that if you're familiar with it, it's going to be much easier to prescribe than something that you have just head about or read about.“
		“I think certainly the more hands-on you can get, I've done a couple or participated in a couple of webinars from the e-mental health stuff probably a year or two ago and that helped with my awareness of things, but my confidence I don’t think improved too much. I think you've got to do them. You've either got to… Use it yourself or see it being used or at least be familiar with what it looks like.”
	Trustworthy source of vetted prescribable apps	“I think having somebody external to narrow down the pool of apps and say this is a decent product, then you don’t mind recommending them in that way.”
		“if it's coming from a reliable source like the university and say these are the apps we think are good quality apps to recommend, then I feel comfortable because there is so much information on the internet and app world, we don't know which is good quality and which is fake.”
	Integration with existing software and workflow	“I think it would be brilliant to have an app that I could use for chronic disease management that actually was integrated, that the patients could potentially put data into that will then be integrated with my software, that would be fantastic.”
		“Certainly, would help to have them integrated into our - the fact that we've prescribed them, into our software, medical software, so that we can just click a button to say recommended whichever app.”
	Visual reminder or cue for prescribable apps	“having those pads in front of me made me think about it, the reminders and having a resource to go to.”
		“I think having something like you did that makes it easy to give them out, that makes it easier and not having too many, just having a few that is quite good.”
	Patients’ capability and attitude towards mHealth apps	“most of the current population, the phone is the one thing that they carry around that they have with them all the time. Instead of - especially them being able to use it as an extra tool, they're useful in the way of treating patients.”
	Proof of benefits of apps as an alternative and or adjunct treatment	“sometimes the apps were very useful for patients who I was aware weren't able to afford other options. So for example, the pelvic floor exercises app would sometimes occur to me when I was talking to patients about the difficulties of accessing physiotherapy due to the cost and it would then prompt me to think, oh yes, actually I have an app that you could try at home without cost.”
		“maybe some data showing that they are received well by patients, I guess. apps showing patient receptiveness and patient engagement”

^a^GP: general practitioner.

^b^mHealth: mobile health.

## Discussion

This study demonstrated that it may be possible to increase the uptake of mHealth app prescription by providing an implementation intervention in the Australian general practice setting. The results demonstrated a total of 1324 app prescriptions by 39 GPs over 4 months and positive feedback from GPs about the intervention. The fortnightly number of apps prescribed per GP more than doubled compared to the prestudy level. However, identified barriers to app prescription uptake were poor knowledge of prescribable apps and insufficient familiarity with the apps to foster confident prescribing habits. Participants identified a need for a reliable prescribable app repository, preferably integrated with their electronic medical systems, and consistent and persistent messaging to increase the knowledge and familiarity of such apps.

The variation in the total individual tally of apps prescribed by participants may reflect differences in their personal digital propensity and flexibility in altering prescribing behavior. The reduction in the monthly app prescriptions after the first month could be related to the timing of the second and third reporting for about half of the participants. These occurred during the Christmas, New Year, and summer holidays in Australia, during which acute conditions dominate GP visits more than chronic conditions, which were the focus of the apps in the intervention.

The app explanation videos had varying effects on app prescription numbers. The results from the qualitative interviews showed that app prescription numbers are primarily dependent on the patient cohort and the prevalence of the conditions for which the intervention apps were intended. Thus, the short explanatory videos were informative but unlikely to be sufficient to influence complex behaviors such as prescribing. Perhaps, it would be more beneficial if video introduction and instructions for mHealth apps were developed for patients and given as part of the app prescription.

This is the first study to test the feasibility of an intervention to increase app prescription in Australian general practice. The overall attrition rate was low, and we analyzed the data as ITT, including those who dropped out of the study. Limitations include lack of access to electronic medical record data of the GP clinics to correlate the prevalence of conditions with the frequency of app prescription within the patient cohort. We aimed to recruit a sample of GPs representative of the national GP cohort; however, our participants’ median age of 40 years was younger than the national average of 50-55 years. Other limitations include a single-group pre-post study design, possible volunteer bias of the participants, and short time frame (4 months). Ideally, a randomized controlled trial should be conducted to establish the long-term effectiveness of the intervention with a large and representative sample for a longer duration. Due to the restrictions of available time and resources, we were unable to achieve this. Future studies should also opt for an electronic version of app prescription to improve sustainability and scalability. Another limitation is the analysis of qualitative data by a single researcher; however, the qualitative data result was a small part of our secondary outcome to primarily answer if the intervention was acceptable and feasible for practicing GPs.

There are few comparable studies of app recommendation in a primary care setting. A trial for an app prescription platform, AppSalut, in Spain involved 32 doctors who made 79 app recommendations in 5 months. Of the three apps they used, a medication adherence app was the most prescribed [4]. It sends the prescribed app to patients as text messages and can monitor and receive data on patients’ use and adherence to the system. In the United States, the Cambridge Health Alliance network of primary care clinics implemented a mental health app dissemination program, in which they evaluated mental health apps, selected 7 apps, and recommended these 7 apps in 12 primary care clinics [5]. Similar to the finding of our study, app prescriptions for anxiety and stress were the most frequently prescribed. An Australian study tested the feasibility of integrating mHealth apps into dietetic practice by asking 5 dietitians to use one chosen app for 12 weeks [6].

All of these studies provided training to the participating health care professionals to educate them about the study apps as well as the electronic systems they needed to use. The qualitative feedback from our participants also included the need for such training. However, because GPs often report being overworked, time-poor, and inundated with different information and offers, it would be challenging to organize out-of-hours training involving many GPs or train dedicated personnel to visit GP clinics during lunch hours, which was suggested by the GPs as a solution. The scalability of such an intervention would pose funding and logistical challenges.

One way to promote the sustainability and scalability of mHealth app integration into clinical practice is to provide an electronic repository of vetted and curated apps for health care professionals. In Australia, the Victoria Department of Health [24], Black Dog Institute [25], and HANDI project by the Royal Australian College of General Practitioners [26] offer small repositories of mHealth apps, but these organizations function under different jurisdictions with no national guideline in place. GPs in our study emphasized the need for a nationally accessible repository of a select few prescribable apps that are relevant to general practice that is safe, reliable, and easy to navigate.

We found that mHealth app prescription is feasible in a general practice setting in Australia by addressing previously identified practical barriers to mHealth app prescription. Our implementation intervention was effective in increasing app prescription. However, the future of app prescription depends on efforts to increase GPs’ knowledge of prescribable apps as well as a dedicated trustworthy app repository for GPs.

## Appendix

Multimedia Appendix 1 Statistical data for intervention video impact.

Multimedia Appendix 2 App prescription pads being used.

## Abbreviations

GP: general practitioner.
HANDI: Handbook of Non-Drug Interventions.
ITT: intention to treat.
mHealth: mobile health.
