# Supplementary material for: mHealth App Prescription in Australian General Practice: Pre-Post Study
Source: JMIR Mhealth Uhealth. 2020 Jun 1;8(6):e16497. doi: 10.2196/16497 (PMC7296416; doi:10.2196/16497)
Supplement: Multimedia Appendix 1 [file mhealth_v8i6e16497_app1.docx]

# Supplementary data 1. Statistical data for intervention video impact calculation

## Statistical methods

Each month for 4 months 40 GPs recorded the number of times they prescribed 6 different apps to patients from their practice. Data was collected for 2 months prior to as well as 2 months after exposure to 1 of 6 videos relating to each of the 6 apps. Each GP was randomly assigned to 1 of the apps. To assess the effect of exposure to the apps Poisson regression was used. Six separate models were fitted with the outcome being counts of monthly prescriptions of the each of the 6 apps. Two explanatory factors were specified: one categorical factor for the month (first, second, third, fourth) included to adjust for any monthly differences in prescriptions due to period effects; and another factor indicating whether the GP had been exposed to the video relating to the app in question. To account for the 4 repeated measures collected on each GP a random intercept was fitted. Over-dispersion was assessed using the generalized Chi-square divided by degrees of freedom where a value around 1 indicates no over-dispersion.

*Global analysis*

To conduct an overall analysis of the effect of video exposure on prescription rates the 6 separate outcomes (1 for each app) were considered as one overall global outcome (note the individual monthly counts were not aggregated). Initially, a Poisson model was fitted with the (categorical) explanatory variables specified being month (1 to 4), exposure to the video (yes/no), video (1 to 6) as well as the interaction between exposure and video. To account for the 24 repeated measures collected on each GP (4 timepoints by 6 apps) a random intercept was fitted. Over-dispersion was assessed using the generalized Chi-square divided by degrees of freedom. Due to evidence of over-dispersion for the Poisson model (Gener. Chi-Square / DF = 2.13) a negative binomial model was subsequently fitted and showed no evidence of over-dispersion (Gener. Chi-Square / DF = 0.98).

## Results

Descriptive data on the counts of monthly prescriptions are tabulated below. One GP did not provide any data and there was some further missing data at months 3 and 4. A global test for an interaction between exposure and video showed strong evidence of heterogeneity (P=0.0002) indicating the treatment effects were different across the 6 videos/apps. Therefore, it is not recommended to report an overall effect of video.

| **Month** | **N Obs** | **Variable** | **N** | **Mean** | **Std Dev** | **Minimum** | **Maximum** |
| --- | --- | --- | --- | --- | --- | --- | --- |
| 1 | 40 | Smiling_Mind  Managn_Deprssn  Tat  Lose_It  CBTi  Quit_Now | 39  39  39  39  39  39 | 3.90  1.69  0.82  1.74  2.23  1.31 | 3.19  1.73  0.94  2.63  2.63  2.27 | 0.00  0.00  0.00  0.00  0.00  0.00 | 16.00  7.00  3.00  15.00  9.00  13.00 |
| 2 | 40 | Smiling_Mind  Managn_Deprssn  Tat  Lose_It  CBTi  Quit_Now | 39  39  39  39  39  39 | 3.05  0.97  0.38  0.85  1.26  0.67 | 2.81  1.29  0.94  1.09  1.41  0.96 | 0.00  0.00  0.00  0.00  0.00  0.00 | 14.00  5.00  5.00  4.00  5.00  4.00 |
| 3 | 40 | Smiling_Mind  Managn_Deprssn  Tat  Lose_It  CBTi  Quit_Now | 37  37  37  37  37  37 | 4.00  1.00  0.65  0.78  1.46  0.76 | 3.61  1.25  1.34  1.08  1.52  0.89 | 0.00  0.00  0.00  0.00  0.00  0.00 | 15.00  4.00  7.00  5.00  5.00  3.00 |
| 4 | 40 | Smiling_Mind  Managn_Deprssn  Tat  Lose_It  CBTi  Quit_Now | 36  36  36  36  36  36 | 3.17  0.72  0.61  0.69  1.44  0.81 | 3.79  1.00  1.08  1.19  1.54  1.17 | 0.00  0.00  0.00  0.00  0.00  0.00 | 17.00  4.00  4.00  5.00  6.00  4.00 |

***Analysis of Smiling Mind prescriptions***

| **Analysis Variable: Smiling_Mind** | | | | | | | |
| --- | --- | --- | --- | --- | --- | --- | --- |
| **Month** | **Video exposure** | **N Obs** | **N** | **Mean** | **Std Dev** | **Minimum** | **Maximum** |
| 1 | 0 | 40 | 39 | 3.90 | 3.19 | 0.00 | 16.00 |
| 2 | 0 | 40 | 39 | 3.05 | 2.81 | 0.00 | 14.00 |
| 3 | 0 | 34 | 32 | 3.69 | 3.49 | 0.00 | 15.00 |
|  | 1 | 6 | 5 | 6.00 | 4.18 | 1.00 | 12.00 |
| 4 | 0 | 34 | 31 | 2.68 | 3.05 | 0.00 | 11.00 |
|  | 1 | 6 | 5 | 6.20 | 6.53 | 0.00 | 17.00 |

Poisson regression showed no evidence of over-dispersion (Gener. Chi-Square / DF = 1.11) and some evidence of an intervention effect where those GPs randomly assigned to the smiling mind app had 61% increase in prescriptions after exposure to the video (95% CI: 6% to 145%, P=0.025). Descriptive statistics in the table above support this finding. The mean number of prescriptions was around 6 after exposure to the smiling mind video compared to means of around 3 to 4 prior to exposure and for those GPs not exposed to the video.

***Analysis of Managing depression prescriptions***

| **Analysis Variable: Managn_Deprssn** | | | | | | | |
| --- | --- | --- | --- | --- | --- | --- | --- |
| **Month** | **Video Exposure** | **N Obs** | **N** | **Mean** | **Std Dev** | **Minimum** | **Maximum** |
| 1 | 0 | 40 | 39 | 1.69 | 1.73 | 0.00 | 7.00 |
| 2 | 0 | 40 | 39 | 0.97 | 1.29 | 0.00 | 5.00 |
| 3 | 0 | 33 | 32 | 1.00 | 1.30 | 0.00 | 4.00 |
|  | 1 | 7 | 5 | 1.00 | 1.00 | 0.00 | 2.00 |
| 4 | 0 | 33 | 31 | 0.77 | 1.02 | 0.00 | 4.00 |
|  | 1 | 7 | 5 | 0.40 | 0.89 | 0.00 | 2.00 |

Poisson regression showed no evidence of over-dispersion (Gener. Chi-Square / DF = 1.02) and no evidence of an intervention effect (-8% 95% CI: -63% to 129%, P=0.86).

***Analysis of Tat prescriptions***

| **Analysis Variable: Tat** | | | | | | | |
| --- | --- | --- | --- | --- | --- | --- | --- |
| **Month** | **Video exposure** | **N Obs** | **N** | **Mean** | **Std Dev** | **Minimum** | **Maximum** |
| 1 | 0 | 40 | 39 | 0.82 | 0.94 | 0.00 | 3.00 |
| 2 | 0 | 40 | 39 | 0.38 | 0.94 | 0.00 | 5.00 |
| 3 | 0 | 33 | 30 | 0.73 | 1.46 | 0.00 | 7.00 |
|  | 1 | 7 | 7 | 0.29 | 0.49 | 0.00 | 1.00 |
| 4 | 0 | 33 | 30 | 0.50 | 0.86 | 0.00 | 3.00 |
|  | 1 | 7 | 6 | 1.17 | 1.83 | 0.00 | 4.00 |

Poisson regression showed no evidence of over-dispersion (Gener. Chi-Square / DF = 1.21) and no evidence of an intervention effect (26% 95% CI: -48% to 203%, P=0.60).

***Analysis of Lose it prescriptions***

| **Analysis Variable: Lose_It** | | | | | | | |
| --- | --- | --- | --- | --- | --- | --- | --- |
| **Month** | **Video exposure** | **N Obs** | **N** | **Mean** | **Std Dev** | **Minimum** | **Maximum** |
| 1 | 0 | 40 | 39 | 1.74 | 2.63 | 0.00 | 15.00 |
| 2 | 0 | 40 | 39 | 0.85 | 1.09 | 0.00 | 4.00 |
| 3 | 0 | 34 | 31 | 0.58 | 0.81 | 0.00 | 3.00 |
|  | 1 | 6 | 6 | 1.83 | 1.72 | 0.00 | 5.00 |
| 4 | 0 | 34 | 30 | 0.60 | 1.00 | 0.00 | 3.00 |
|  | 1 | 6 | 6 | 1.17 | 1.94 | 0.00 | 5.00 |

Poisson regression showed no evidence of over-dispersion (Gener. Chi-Square / DF = 1.13) and some evidence of an intervention effect where those GPs randomly assigned to the lose it app had 144% increase in prescriptions after exposure to the video (95% CI: 21% to 390%, P=0.013). Descriptive statistics in the table above support this finding.

***Analysis of CBT-i Coach prescriptions***

| **Analysis Variable: CBTi** | | | | | | | |
| --- | --- | --- | --- | --- | --- | --- | --- |
| **Month** | **Video exposure** | **N Obs** | **N** | **Mean** | **Std Dev** | **Minimum** | **Maximum** |
| 1 | 0 | 40 | 39 | 2.23 | 2.63 | 0.00 | 9.00 |
| 2 | 0 | 40 | 39 | 1.26 | 1.41 | 0.00 | 5.00 |
| 3 | 0 | 33 | 30 | 1.47 | 1.50 | 0.00 | 5.00 |
|  | 1 | 7 | 7 | 1.43 | 1.72 | 0.00 | 5.00 |
| 4 | 0 | 33 | 29 | 1.28 | 1.49 | 0.00 | 6.00 |
|  | 1 | 7 | 7 | 2.14 | 1.68 | 0.00 | 4.00 |

Poisson regression showed no evidence of over-dispersion (Gener. Chi-Square / DF = 1.17) and no evidence of an intervention effect (43% 95% CI: -19% to 154%, P=0.22).

***Analysis of Quit now prescriptions***

| **Analysis Variable: Quit_Now** | | | | | | | |
| --- | --- | --- | --- | --- | --- | --- | --- |
| **Month** | **Video exposure** | **N Obs** | **N** | **Mean** | **Std Dev** | **Minimum** | **Maximum** |
| 1 | 0 | 40 | 39 | 1.31 | 2.27 | 0.00 | 13.00 |
| 2 | 0 | 40 | 39 | 0.67 | 0.96 | 0.00 | 4.00 |
| 3 | 0 | 33 | 30 | 0.83 | 0.91 | 0.00 | 3.00 |
|  | 1 | 7 | 7 | 0.43 | 0.79 | 0.00 | 2.00 |
| 4 | 0 | 33 | 29 | 0.76 | 1.09 | 0.00 | 4.00 |
|  | 1 | 7 | 7 | 1.00 | 1.53 | 0.00 | 4.00 |

Poisson regression showed no evidence of over-dispersion (Gener. Chi-Square / DF = 0.83) and no evidence of an intervention effect (8% 95% CI: -53% to 150%, P=0.86).

***Overall results: Mean number of app prescription per GP before and after exposure to intervention video at each month (mean, (95% CI))***

| Month  Apps | 1 | 2 | 3 | 4 |
| --- | --- | --- | --- | --- |
| Smiling mind video | 3.9 (2.9, 4.9) | 3.05 (2.17, 3.93) | 6 (2.66, 9.34) | 6.2 (0.48, 11.92) |
| No video |  |  | 3.69 (2.48, 4.9) | 2.68 (1.6, 3.75) |
| Managing Depression video | 1.69 (1.15, 2.23) | 0.97 (0.56, 1.37) | 1 (0.12, 1.88) | 0.4 (-0.38, 1.18) |
| No video |  |  | 1 (0.55, 1.45) | 0.77 (0.41, 1.13) |
| Tat video | 0.82 (0.53, 1.12) | 0.38 (0.09, 0.68) | 0.29 (-0.07, 0.65) | 1.17 (-0.29, 2.63) |
| No video |  |  | 0.73 (0.21, 1.25) | 0.5 (0.19, 0.81) |
| Lose-It! video | 1.74 (0.91, 2.57) | 0.85 (0.51, 1.19) | 1.83 (0.45, 3.2) | 1.17 (-0.38, 2.72) |
| No video |  |  | 0.58 (0.29, 0.87) | 0.6 (0.24, 0.96) |
| CBT-i Coach video | 2.23 (1.4, 3.06) | 1.26 (0.82, 1.70) | 1.43 (0.16, 2.7) | 2.14 (0.9, 3.38) |
| No video |  |  | 1.47 (0.93, 2) | 1.28 (0.74, 1.82) |
| My QuitBuddy video | 1.31 (0.6, 2) | 0.67 (0.37, 0.97) | 0.43 (-0.15, 1.02) | 1 (-0.13, 2.13) |
| No video |  |  | 0.83 (0.5, 1.16) | 0.76 (0.36, 1.16) |
